# Properties and Applications of PDMS for Biomedical Engineering: A Review

**DOI:** 10.3390/jfb13010002

**Published:** 2021-12-21

**Authors:** Inês Miranda, Andrews Souza, Paulo Sousa, João Ribeiro, Elisabete M. S. Castanheira, Rui Lima, Graça Minas

**Affiliations:** 1 Center for MicroElectromechanical Systems (CMEMS-UMinho), Campus de Azurém, University of Minho, 4800-058 Guimaraes, Portugal; ines_sofia_miranda@outlook.com (I.M.); psousa@dei.uminho.pt (P.S.); gminas@dei.uminho.pt (G.M.); 2 MEtRICs, Mechanical Engineering Department, Campus de Azurém, University of Minho, 4800-058 Guimaraes, Portugal; andrewsv81@gmail.com; 3 Centro de Investigação de Montanha (CIMO), Campus de Santa Apolónia, Instituto Politécnico de Bragança, 5300-253 Braganca, Portugal; jribeiro@ipb.pt; 4 Centre of Physics of Minho and Porto Universities (CF-UM-UP), Campus de Gualtar, University of Minho, 4710-057 Braga, Portugal; ecoutinho@fisica.uminho.pt; 5 CEFT, Faculdade de Engenharia da Universidade do Porto (FEUP), Rua Roberto Frias, 4200-465 Porto, Portugal

**Keywords:** polydimethylsiloxane, PDMS properties, PDMS applications, microfluidics, biomedical engineering

## Abstract

Polydimethylsiloxane (PDMS) is an elastomer with excellent optical, electrical and mechanical properties, which makes it well-suited for several engineering applications. Due to its biocompatibility, PDMS is widely used for biomedical purposes. This widespread use has also led to the massification of the soft-lithography technique, introduced for facilitating the rapid prototyping of micro and nanostructures using elastomeric materials, most notably PDMS. This technique has allowed advances in microfluidic, electronic and biomedical fields. In this review, an overview of the properties of PDMS and some of its commonly used treatments, aiming at the suitability to those fields’ needs, are presented. Applications such as microchips in the biomedical field, replication of cardiovascular flow and medical implants are also reviewed.

## 1. Introduction

Polydimethylsiloxane (PDMS) is an elastomeric polymer with interesting properties for biomedical applications, including physiological indifference, excellent resistance to biodegradation, biocompatibility, chemical stability, gas permeability, good mechanical properties, excellent optical transparency and simple fabrication by replica moulding [1,2,3,4,5]. Due to these characteristics, PDMS has been widely used in micropumps [6], catheter surfaces [7], dressings and bandages [8], microvalves [9], optical systems [10,11], in the in vitro study of diseases [12,13], in implants [14,15], in microfluidics and photonics [16,17,18,19]. Moreover, soft-lithography technology has driven the use of PDMS in microelectromechanical systems (MEMS) applications and in microfluidic components [17,18,20]. Soft-lithography techniques such as micro-contact printing, replica moulding, micro-transfer moulding, micro-moulding in capillaries and solvent-assisted micro-moulding usually require the use of PDMS to create an elastomeric stamp or mould that incorporates nano- and microstructures for the transfer of patterns onto a subsequent substrate [18,21].

MEMS are approaches that use electronic and mechanical technologies to deal with biomedical problems on the micro-scale [22]. MEMS-based devices have been widely used in the biomedical area for applications such as diagnostics and therapeutics. These systems can be microsensors or microtransducers, and are helpful in areas such as physics, mechanics, electronics and biomedicals, as they can provide very precise and fast results [23]. The investigation and improvement of already existing MEMS are more and more common. As they are increasingly commercialized, the necessity to find processes and materials that enable mass production while reducing cost has emerged [21]. MEMS are traditionally silicon-based and the pursuit for a more biologically friendly material is needed. Polymers allow rapid prototyping and mass production techniques as well as having a lower cost in relation to silicon, making them particularly attractive for the development of MEMS [21]. Photolithography is the most commonly used technique in microfabrication, however, this method is expensive [24]. With the introduction of polymers in microsystems, new manufacturing techniques have been studied, such as soft-lithography, which can be a cheaper method comparatively to photolithography, even when a costly mould is needed for patterning; once a mould is created, it can be reused several times [20]. Additionally, there are alternatives which are attempting to reduce the cost of the moulds, relying on cleanroom less approaches [25]. Candidate polymers for the production of MEMS are polycarbonate (PC), polymethylmethacrylate (PMMA), polyvinylchloride (PVC), polyethylene (PE) and PDMS [21].

Additionally, PDMS is the most commonly used material in the manufacturing of microfluidic devices, which are an important technology for the development of systems such as drug delivery, DNA sequencing, clinical diagnostics, point of care testing and chemical synthesis [26]. The used materials in these systems should be biocompatible, optically transparent and provide fast prototyping and low fabrication cost [27], features found in PDMS.

In addition to applications in microfluidics, PDMS has been widely used in the fabrication of biomodels (flow phantom) for the in vitro hemodynamic study of diseases such as aneurysms and stenosis [28,29,30,31]. The biomodels developed in PDMS allow good replicability of the lumen of the arteries and good transparency, being ideal for the application of optical techniques of micro particle image velocimetry (micro-PIV), particle image velocimetry (PIV), particle tracking velocimetry (PTV) and non-evasive techniques [32,33,34]. These experimental tests have provided a greater understanding of these pathologies, validated numerical techniques and tested medical devices such as stents [35,36,37].

PDMS has also been investigated in the field of medical implants [38,39,40,41,42]. These types of implants are usually made with titanium or its alloys; however, such materials do not allow a good osseointegration [39]. In order to overcome this limitation, PDMS has been studied to produce coatings with microscale features that help the bonding between the implant and the bone. The main characteristics for its use in implants are its high biocompatibility, excellent resistance to biodegradation and flexibility, which makes PDMS one of the most successful polymers in implanted devices, presenting only mild foreign body reactions [43,44,45]. Common applications include cardiac pacemakers, cuff and book electrodes in the PNS, cochlear implants, bladder and pain controllers and planar electrode arrays in the CNS [45,46].

In this review, research on PDMS properties, their fabrication processes and their characterization methods are reported. Moreover, their use in MEMS applications, microfluidics, medical implants and hemodynamic studies is investigated. Written in a concise, but complete manner, we believe that this manuscript joins together the main advantages, disadvantages and challenges of PMDS when biomedical applications are needed and, therefore, can be extremely useful for researchers looking to learn about this biomaterial and its applicability in this biomedical field.

## 2. PDMS Properties

Silicon, glass and polymers are the typical materials used for micro devices fabrication: silicon, because of its thermal conductivity and the availability of advanced fabrication technologies; glass, mainly due to its transparency; polymers, because of its low cost, optical transparency and flexibility. Compared to glass and silicon, PDMS turns out to be the most promising elastomer, because the other two materials have a high manufacturing cost, require greater labour intensity and are rigid in nature. The variable elasticity of PDMS in medical applications is also favourable; its modulus of elasticity is 1–3 MPa (compared to ~50 GPa of glass) [2,47]. PDMS is also chemically inert, thermally stable, permeable to gases, simple to handle and manipulate, exhibits isotropic and homogeneous properties and can replicate submicron features to develop microstructures [19,21,48]. Additionally, this elastomer is optically transparent, can work as a thermal and electrical insulator and degrades quickly in the natural environment [49]. PDMS presents a hyperelastic behaviour, which is the ability of a material to undergo large deformations before rupture [50]. This characteristic is also found in biological tissues and, for that reason, PDMS is a well-suited material to mimic, for example, blood vessels [49]. Another characteristic of this elastomer is its biocompatibility, which means that PDMS is compatible with biologic tissues [49]. PDMS presents a transmittance up to 90% for the wavelength from 390 nm to 780 nm [51,52,53] and, due to this characteristic, PDMS-based microsystems allow the direct observation of the mimicked blood flow inside the mimicked vessels and the integration of optical detection systems, hence playing an important role in this field.

With the purpose of extending the lifespan of a chip, PDMS is used to embed or encapsulate electronic components by casting. Due to its thermal and electrical insulation capability, PDMS protects the components from environmental factors and mechanical shock within a large temperature range (−50–200 °C) [23,48]. In Table 1, some physical properties of PDMS are listed.

Despite these advantages, PDMS has some properties that can present a limitation in some applications. Due to its CH_3_ groups, PDMS presents a hydrophobic surface (contact angle with water ~108° ± 7°) [62,64,65], often limiting its application in solutions composed of biological samples [66]. Additionally, PDMS tends to swell when combined with certain reagents [17,48]. In some applications, the absorption of small molecules flowing through the channels makes it difficult to quantitatively analyse experiments in proteomic drug discovery and cell culture [67,68]. In microchannels, the hydrophobicity of PDMS generates complications that include impedance to the flow of polar liquids, which makes it difficult to wet its surface with aqueous solvents [49]. On the other hand, much effort has been made to make the PDMS surface hydrophilic and resistant to protein adsorption [19,69,70,71,72,73].

Strategies employed in attempting to solve PDMS hydrophobicity include surface activation methods such as: oxygen plasma; UV/ozone treatments; corona discharges, which are widely used for PDMS surface oxidation to promote microchannel wettability. The main benefits of these methods are the short treatment time and easy operation; however, the PDMS surface recovers its hydrophobicity when in contact with air within a few minutes [74,75,76]. The hydrophilic treatments and some examples are discussed further in Section 5.

Another method is physisorption, which is a simple and efficient approach that relies on surface hydrophobic or electrostatic interactions. This method includes the following techniques: layer-by-layer deposition; non-ionic surfactants; charged polymers. The disadvantages are the lack of covalent bonds between PDMS and surface modifiers, which lead to the loss of modifiers quickly through desorption [77,78,79].

In order to improve the difficulties encountered in physisorption, chemical modification methods allow for maintaining a long-term stability of the modified surface. These methods include: chemical vapor deposition, surface segregation and self-assembled monolayers, silanization, and polymer brushes via grafting methods [1,62,80,81,82].

Adding waxes such as paraffin or beeswax to PDMS has been demonstrated to potentially increase the corrosion resistance, hydrophobicity and thermal and optical properties of PDMS, which is useful in applications such as sensors, wearable devices and superhydrophobic coating [83].

Although the methods listed above have been successful in improving the hydrophilicity of the PDMS surface, they have some limitations, such as chemical instability, the need for specific equipment, limited manufacturing process for large scale and some methods cause loss of transparency, loss of mechanical properties and do not provide the hydrophilic surface for a long period of time [62]. Considering these facts, the work of Gökaltun et al. [84] presents a simplified method of easy manufacture, which uses copolymers composed of poly(ethylene glycol) and PDMS segments (PDMS-PEG) to reduce the hydrophobicity of PDMS without changing its transparency, biocompatibility and mechanical properties, with a durability of 20 months.

## 3. PDMS Manufacturing Process

Sylgard^®^ 184 Silicone Elastomer Kit is the most used commercial PDMS. It consists of a monomer and a curing agent, which are usually combined at a weight ratio of 10:1. The compound is mixed and then degassed with a desiccator in order to prevent the formation of micro-bubbles. The PDMS solution is poured over the master mould and then cured in the oven [23]. The curing time depends on the temperature of the oven and on the size of the PDMS sample. The higher the hardening temperature, the less time it will take for the PDMS to cure. After the curing process, the piece is taken out of the mould [57]. Note that, for very specific applications and complex geometries, it is usually advised to perform the curing process at room temperature for at least 48 h [55,85]. In Table 2 are listed curing times and temperatures recommended by the manufacturer.

The monomer and the curing agent can be mixed at a different ratio besides the 10:1 [86] and, as a consequence, some properties change, namely, mechanical [87], optical [88] and gas permeability [89]. Mixing at a higher ratio of cure agent results in a faster hardening time, in a less sticky cured PDMS and in a more fragile PDMS sample. In contrast, mixing with less cure agent results in a longer hardening time, in a stickier cured PDMS and in better mechanical properties. Khanafer et al. [87] found that elastic modulus increases as the mixing ratios increase up to 9:1, after which the elastic modulus starts to decrease as the mixing ratio continues to increase.

## 4. Methods to Characterize PDMS

A wide range of tests are performed to characterize elastomers. Some common tests are scanning electron microscopy, gravimetry, goniometry, nanoindentation, tensile test, X-ray photoelectron spectroscopy and Fourier Transform infrared spectroscopy [21]:

- Scanning electron microscopy (SEM) allows thickness measurement and qualitative characterization of PDMS samples [18,42,90,91,92];
- Gravimetry is a method based on gravitational techniques to quantify changes in PDMS sample weight. For example, this method is useful when it is needed to verify if there was or not degradation of the PDMS after chemical immersion [93];
- In order to obtain information on surface hydrophilicity, a goniometry test is performed. Micro water droplets are dropped on the PDMS surface and then the contact angle is measured. This technique allows for verification of if there was or not a change in the wettability of the PDMS after certain treatments [19,39,42,94];
- Nanoindentation offers the possibility of studying mechanical properties of the outermost layer of PDMS, which is susceptible to destruction due to different treatments, such as UV irradiation [95];
- Tensile testing allows Young Modulus measurement of PDMS. The Young Modulus can be affected by treatments that may be applied to PDMS, by hardening temperature and time, and by the mixing ratio used to fabricate the PDMS samples [42,96,97];
- X-ray photoelectron spectroscopy (XPS) is a technique based on the photoelectric effect, which allows identification of the elemental composition of the material. This method is useful when it is needed to verify if any changes in surface composition occurred after the PDMS received any treatment [38,39,98];
- Fourier Transform infrared spectroscopy (FTIR) is a method used to obtain the infrared spectrum of absorption or transmission of the PDMS sample. This technique allows examination of the effect of some treatment on the cross-linking of PDMS [38,42,99].

## 5. PDMS Microfabrication

PDMS is patterned through commonly used microfabrication techniques, such as soft-lithography and spin coating. However, especially due to its hydrophobic nature, some of the techniques must be employed alongside with hydrophilic treatments, such as oxygen plasma. Soft-lithography, which is a group of techniques that use patterned elastomers as stamp, mould or mask to generate micropatterns, was developed to allow processing elastomers [100]. However, the fabrication of the most microfluidic devices still relies on photolithography for fabricating SU-8 masters that usually serve as the PDMS mould [20]. Photolithography is a microfabrication technique used to process photoresists, commonly employed in CMOS microelectronics fabrication [101]. The soft-lithography can be performed in several types, such as microcontact printing (µCP), replica moulding (REM), micro-transfer moulding (µTM), micro-moulding in capillaries (MIMIC), solvent-assisted micro-moulding (SAMIM), phase-shift photolithography, cast moulding, embossing and injection. Some of these techniques are briefly described below [100]:

1. Microcontact printing: uses the relief pattern on the surface of a PDMS stamp to form patterns of self-assembled monolayers (SAMs) on the surfaces of substrates by contact;
2. Replica moulding: replicates the relief pattern on the surface of a PDMS mould by using this structure as a mould for forming structures in a second UV-curable (or thermally curable) prepolymer;
3. Micro-transfer moulding: a thin layer of liquid prepolymer is applied to the patterned surface of a PDMS mould. It is then placed in contact with the surface of a substrate and the liquid prepolymer is cured to a solid. After peeling off the mould, a patterned micro-structure is left on the surface of the substrate;
4. Micro-moulding in capillaries: a PDMS mould is placed on the surface of a substrate to form a network of empty channels between them. The channels are filled with a low viscosity prepolymer, which is then cured to a solid. The mould is removed and a patterned micro-structure is left on the surface of the substrate;
5. Solvent-assisted micro-moulding: a PDMS mould is wetted with a solvent, and it is placed in contact with a substrate (typically an organic polymer). The solvent starts to dissolve the substrate into a fluid or gel that is moulded against the relief structures in the mould. When the fluid solidifies, it forms a pattern relief structure complementary to that in the surface of the mould.

The soft-lithography process begins with the preparation of the elastomeric stamp or the mould by cast moulding. Most of the time, cast moulding implies the use of photolithographic techniques to fabricate the master. PDMS is the most widely used elastomer for this process because of its outstanding properties: low interfacial free energy, it does not swell with humidity, good thermal stability, optical transparency, isotropy and homogeneity [100].

Additionally, spin coating is a common microfabrication method for producing polymer films of controlled and uniform thickness. In this process, a liquid film is spread by centrifugal force onto a rotating substrate. This technique is commonly used for deposition of polymer resist layers in the photolithographic processing of a master mould. It is formulation dependent: increased amounts of cross-linker agent in the formulation decrease film thickness [21,102].

The hydrophobic nature of PDMS brings, in some cases, limitations in the microfabrication processes. There are applications, such as cell culture, immunoassay and biomolecule separation, where the modification of the hydrophobic surface of native PDMS to a hydrophilic surface is indispensable. For example, when endothelial cell seeding is needed, hydrophilic modification of the PDMS surface is indispensable for a successful seeding [103]. However, it is important that the hydrophilic treatment does not affect its transparency, as transparency is a key property that makes PDMS the material of choice for certain applications. Oxygen plasma is the most employed treatment that leads to an increase in PDMS surface hydrophilicity because of its short treatment time, its easy operation and that it does not affect the PDMS transparency [21,65,104,105]. However, this treatment is also known for losing its effects within minutes after exposure to air. For this reason, a variety of well-studied treatments have emerged for this purpose [19,69,70,71,72,73]. Additionally, some articles reported that oxygen plasma may damage PDMS surface [62,106] and, therefore, Shin et al. reported three different treatments that do not require oxygen plasma pre-treatment, including Teflon coating, commercially available water-repellents and perfluorodecyltrichlorosilane (FDTS) [107]. The authors showed that the Teflon and the water-repellent decreased the hydrophobicity of PDMS with great chemical stability and without significantly affecting its transparency. UV/ozone treatments and corona discharge are also commonly employed hydrophilic treatments; however, as with oxygen plasma treatment, PDMS quickly recovers its hydrophobicity [62]. There have been efforts to improve some of these treatments; however, the best way to achieve an effective and long-lasting treatment seems to be the combination of a surface activation with a covalent surface functionalization [19,106]. For example, Zhao et al. [108] proposed a method where PDMS is firstly activated by oxygen plasma treatment and then it is coated with a zwitterionic poly(methacrylate) copolymer (PMGT). This method allowed for decreasing the water contact angle (WCA) of native PDMS from 108° to 30°, with a duration of at least 200 h. Further, Zhou et al. [19] suggest a combination of gas-phase with wet chemical methods in order to achieve a better surface stability in a shorter treatment time. Examples of these treatments are the combination of UV or plasma treatment and silanization, the combination of UV or plasma treatment and graft polymerization and the combination of plasma treatment and layer-by-layer (LBL) assembly.

Sterilization is a required procedure for most biomedical applications. In some cases, this process must be done alongside the microfabrication process. There are three mainly used sterilization methods: cleaning with ethanol, ultraviolet light exposure and the steam autoclave procedure. Sterilization does not significantly affect PDMS hydrophobicity. However, steam autoclaving increases the storage modulus and ultimate tensile stress [21,109,110,111,112,113,114].

## 6. PDMS Applications

### 6.1. PDMS-Based Microchip

**Figure 1:**
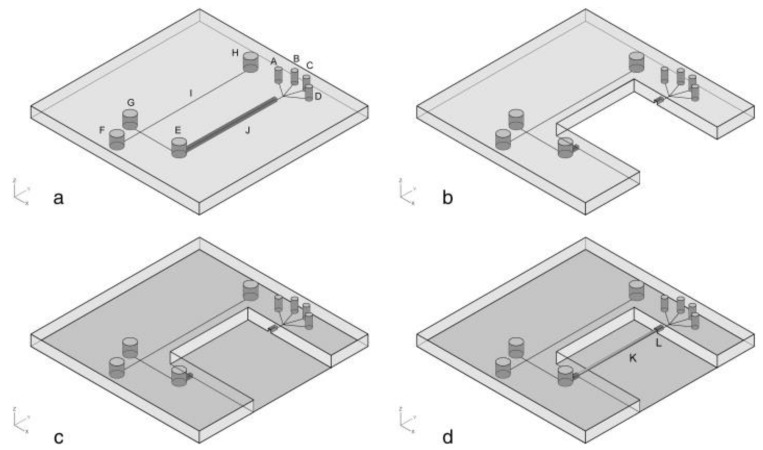
Schematic illustration of the fabrication for glass/PDMS microchip: (**a**) PDMS layer fabricated by replica moulding; (**b**) part of SPE channel in PDMS layer was cut off; (**c**) PDMS layer was sealed with the thin glass cover slip; (**d**) MISPE monolithic capillary column was coupled with glass/PDMS chip to form the final chip. A, B, C, D: holes, E: sample reservoir, F: buffer reservoir, G: sample waste reservoir, H: buffer waste reservoir, I: separation channel, J: SPE channel, K: MISPE monolithic capillary column and L: epoxy glue. Reprinted with permission from reference [115]. Copyright 2020 Elsevier.

Microfluidic devices have been widely studied and developed and, in order to take them to the market, they must be low-cost and capable of mass production. The use of PDMS to fabricate these devices makes it possible to achieve those goals. Nowadays, there are a variety of PDMS-based microchips that have been developed, most of them alongside glass [115,116,117,118,119,120,121]. The combination of PDMS and glass has been employed with great results. In Figure 1 is presented a schematic illustration of the fabrication of a glass/PDMS microchip. For example, Schöning et al. [116] developed a PDMS/glass separation microchip, based on typical semiconductor-compatible production methods, and which provides a simplification of the electrophoresis-based biosensor set-up.

Temperature gradient generation is a commonly used process in microfluidics. Ha et al. [122] presented a PDMS microchip that allows temperature gradient generation using sound waves as a heating mechanism. The use of PDMS allowed the fabrication of a transparent, dynamic, inexpensive and easy-to-fabricate system.

The hydrophobic nature of PDMS usually brings issues to the microchips. Qiang Niu et al. [120] developed a PDMS/glass microchip for PCR; however, the team came across the formation of bubbles on the PDMS surface during the sample loading. To overcome this, they implemented an irreversible bonding and sealing between the glass and PDMS. Additionally, protein adsorption occurred on the chip surface, which was overcome by treating the surface with BSA (Bovine Serum Albumin). Table 3 presents a list of PDMS-based microchips, as well as the motivations that led the authors to use PDMS in their devices.

### 6.2. PDMS Biomodels for Hemodynamic Studies

As mentioned above, PDMS can be very useful in the fabrication of microchips that allow analysing samples. Reports on the use of this material for the replication of cardiovascular flow are also found in the literature. This type of application allows a better understanding and study of cardiovascular diseases, such as aneurysms.

An aneurysm is characterized by artery wall weakness, which can lead to artery rupture and, consequently, to death. Hemodynamic studies have been done to understand aneurysms; however, they cannot explain the mechanical effects on the expansion of the aneurysm walls [49]. To understand these mechanical effects, studies were conducted where an intercranial aneurysm model was developed using PDMS to simulate the mechanical behaviour of blood vessels [49,123]. PDMS is a well-suited material for this purpose due to its hyperelastic behaviour, which is very similar to that of blood vessels, and the ability to make circular microchannels. Another advantage of using PDMS is that it is transparent, which facilitates monitoring of the blood flow. Additionally, recent studies show that is possible to seed a culture of endothelial cells on the microchannels’ walls, which allows the creation of a very similar environment to that found in microcirculation [90,124,125]. Lima et al. [126] proposed a microfluidic device containing rectangular microchannels in PDMS, where in vitro blood flow measurements were conducted by means of a confocal micro-PIV system. The authors demonstrated that, by using soft-lithography, it is possible to produce precise and reproductible rectangular microchannels and to perform detailed blood flow studies. The same authors have performed a similar study, this time by using circular PDMS microchannels [127]. Although there are already several studies using circular microchannels [127,128,129,130], the majority of the PDMS microchannels used to study in vitro blood flow phenomena have rectangular cross sections. Hence, by using rectangular PDMS microchannels, several research works have been performed on different kinds of constrictions to study the deformability behaviour of blood cells [131,132,133,134,135,136] and air bubbles [137,138]. Cell deformability is a biomarker which can be used to distinguish between healthy and diseased cells. Microfluidic models have been developed in order to better understand and, consequently, diagnose diseases such as malaria [139,140], cancer [141,142] and end-stage kidney disease [143]. Most studies aim to better understand red blood cells (RBC); however, Rodrigues et al. [144] developed a novel integrative microfluidic device which is capable of assessing the deformation index of both white blood cells (WBC) and RBC. The same author also presented a microfluidic tool to study the hemocompatibility of nanoparticles synthesized for theragnostic applications [145]. Additionally, by using microchannels having bifurcations and confluences, several studies have been carried out to better understand the influence of these complex geometries on blood flow behaviour [146,147,148,149,150,151,152].

**Figure 2:**
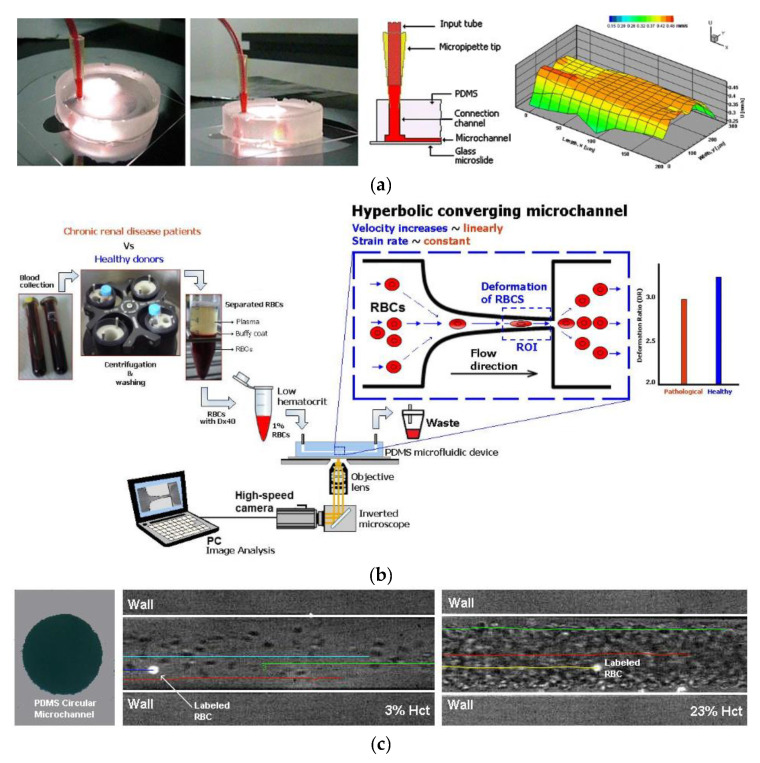
Example of PDMS biomodels for hemodynamic studies: (**a**) rectangular PDMS microchannel to study in vitro blood and ensemble velocity profiles (U) obtained in the middle plane by means of a confocal micro-PIV system (adapted from [126]); (**b**) schematic diagram of the blood collection and cells deformability tests in PDMS microfluidic device (from [143]); (**c**) circular PDMS microchannels to study in vitro blood behavior (adapted from [152]).

Rectangular microchannels are the most common geometry obtained by soft-lithography. However, this kind of geometry can lead to some erratic measurements because the shear stress imposed on the cell is different and, consequently, the pressure build-up in the channel is not the same as if it were built-up in a circular section [124]. Hence, studies have been conducted to establish methods that allow the construction of circular microchannels of PDMS. For example, Fiddes et al. [124] proposed a method which begins by fabricating rectangular microchannels using soft-lithography techniques, followed by the introduction of a stream gas and a solution of the silicone oligomer in an organic solvent. Then, through the polymerization of the oligomer and the removal of the solvent, the authors demonstrated the ability to control the shape and the diameter of the microchannel’s cross-section. Additionally, Choi et al. [90] showed that, combining soft-lithography techniques with the reflow phenomenon of a positive photoresist, it is possible to generate circular PDMS microfluidic channels. In Figure 2 are presented some examples of biomodels for hemodynamic studies.

It is important to notice that, despite the ability to mimic the cardiovascular vessels behaviour through PDMS microchannels, there would always be missing points. For that reason, it is of great importance to combine the PDMS micro devices with well-suited measurement techniques. Rodrigues et al. [123] proposed the use of the Digital Image Correlation (DIC) method, which proved to be suitable to study small displacements happening in in vitro models. A summary of the advantages and limitations of some of the methods used to fabricate microchannels is presented in Table 4.

### 6.3. PDMS-Based Blood Analogues

Blood analogues are fluids commonly used to perform hemodynamic experiments due mainly to safety problems related to the use of real blood in these experiments. Initially, blood analogues were simple fluids composed by mixtures of glycerol and water or by xanthan gum diluted in glycerine and/or water [153,154]. However, by using these kinds of blood analogues, it is not possible to study different kinds of flow phenomena that happen at the micro scale level, such as the cell-free layer, plasma skimming and cell margination [101,155,156]. These microcirculation phenomena do not happen by using blood analogue fluids without solid elements, such as microparticles and microcapsules. Hence, during the past years, several works have been developing different kinds of particulate blood analogue fluids containing microparticles with varying stiffness, shape and size for biomedical applications [157,158,159,160,161,162,163,164,165,166].

**Figure 3:**
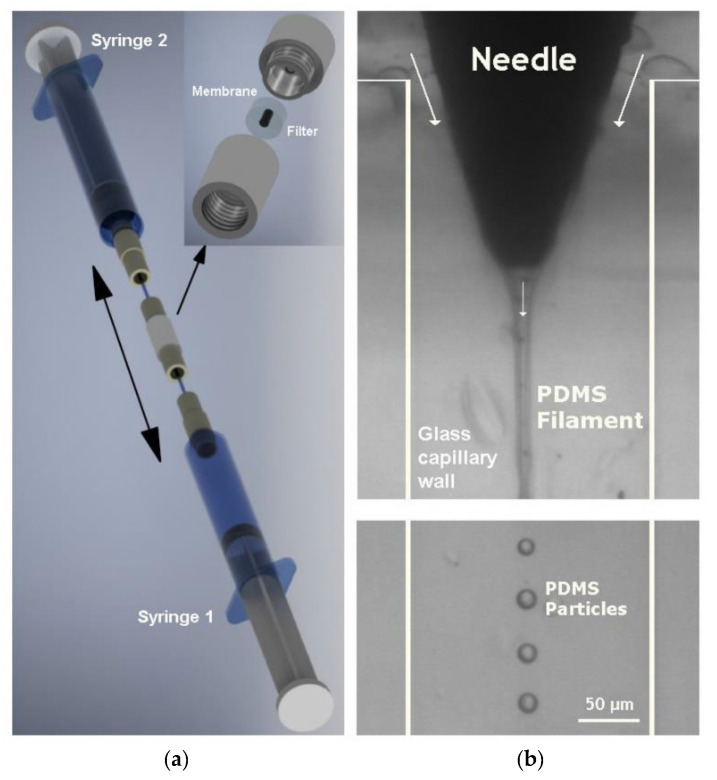
Flexible PDMS microparticles to be used in blood analogue fluids for biomedical applications, produced by (**a**) a two-syringe membrane emulsification technique (adapted from [171]); (**b**) a flow-focusing technique (adapted from [173]).

Due to its unique mechanical properties, PDMS has also been used to produce flexible microparticles to be used in blood analogue fluids. Recently, Muñoz-Sánchez et al. [167] proposed a flow-focusing technique to produce flexible PDMS microparticles for biomedical applications (Figure 3). The PDMS microparticles were produced by using different kinds of ratios (base/curing agent), and rheological measurements performed with a ratio of 6:4 have demonstrated the ability to reproduce the steady shear viscosity curve of ovine RBCs suspended in Dextran 40 [167,168,169]. Although it is possible to produce flexible PDMS microparticles with a high degree of monodispersity by using the flow-focusing technique, the production rate is relatively low. In order to overcome this limitation, Choi et al. [170] and Lopez et al. [171] have proposed a simple emulsification technique to obtain a mass production of PDMS microparticles. More recently, Carneiro et al. [172] have developed another method, based on a multi-stage membrane emulsification process, to obtain high throughput production of PDMS microparticles. The development of blood analogue fluids with PDMS microparticles that mimic the behaviour of RBCs is still at an early stage of development. The most critical challenges that need to be solved are the mass production of monodisperse PDMS microparticles, stiffness, aggregation and fast agglomeration of the PDMS particles within microchannels with complex geometries, such as constrictions and bifurcations.

### 6.4. PDMS-Based Coatings for Medical Implants

PDMS has been widely studied to integrate medical implants, especially due to its biocompatibility. Such implants are usually fabricated with biomedical grade metals (e.g., tantalum, zirconium, niobium), as well as titanium and its alloys [174]. However, these present some limitations concerning blood compatibility, bone conductivity and bioactivity [38]. When developing an implant, some important aspects should be taken in consideration: biocompatibility, osseointegration, corrosion resistance and micro-invasiveness. Osseointegration is related to the effective linkage between the metal and the bone. A weak bonding can lead to the formation of biofilms on implants, which can cause infections. Recent studies have demonstrated that the surface modification of implants, in order to achieve nano-/microscale features, brings great advantages concerning osseointegration [39].

The creation of microscale features on ceramics or polymers is simpler than on metal. Considering the fact that PDMS allows the fabrication of hydrophobic and smooth surfaces has led to their use for developing coatings that help in the osseointegration of implants [38,39,40,41,42]. Rossi de Aguiar et al. [38] studied a sol-gel coating based on PDMS for metallic surfaces such as titanium and stainless steel. The authors demonstrated that the hydrophobic nature of PDMS allows the formation of an anti-biofouling surface, preventing the bacterial adhesion. Additionally, Tran et al. [39] developed a coating that involved the hydrolysis and co-condensation of PDMS and tantalum (Ta) ethoxide to produce tantalum oxide. This PDMS hybrid material has biocompatibility and corrosion resistance properties, which allowed a great osseointegration. The integration of nanoparticles, such as CuO, has been proven to improve the antibacterial characteristic of PDMS-based coatings, as demonstrated by Tavakoli et al. [42]. Table 5 comprises a list of some PDMS-based coatings that have been developed in the past years.

## 7. Conclusions and Further Perspectives

Microchips for biomedical applications are devices that allow monitoring and analysis of samples. The use of PDMS in these devices offers great advantages such as optical transparency, being easy-to-manufacture and having a low-cost, which are important requirements when fabricating microchips. Additionally, the permeability to gases is a unique advantage to culture living cells in closed microchannels, a task that is extremely complex to achieve in glass microchannels. However, the hydrophobic nature of PDMS brings some limitations during the fabrication and flow transport phenomena, especially for biological applications. Developments of treatments that contradict the hydrophobic property have been made and these limitations are easily overcome by applying simple and fast hydrophilic treatments to PDMS. The lack of industrial processes to manufacture PDMS is still an issue. There are already methods that allow good replications of microfabricated PDMS; however, they are far from an industrial scale.

Replication of the cardiovascular system using PDMS microchannels is on a good path to be an application well-suited for the study of cardiovascular diseases. The hyper-elastic behaviour and transparency are great advantages that make PDMS the chosen material in these types of applications. Herein, the hydrophobic nature of PDMS can be a limitation as well, in the blood flow itself but also when it is intended to grow endothelial cell cultures on its walls.

Additionally, PDMS plays an important role in medical implant applications, especially due to its biocompatibility and hydrophobic nature. These characteristics allow the production of antibacterial coatings for implants, which is a requirement when developing implants. PDMS also allows the production of smooth surfaces through processes of microfabrication that help in the osseointegration of the implant in the body. Although PDMS coatings are already available on the market, there are more developments that can be made to increase their features and durability.

It is interesting to note that the hydrophobic nature of PDMS can be a limitation in some applications, such as microchips and microchannel fabrication, but a great advantage in others, such as in implant coatings, solar panels and face masks.

In summary, PDMS opens a wide range of possibilities to make great developments in biomedical applications. With regards to further work, it is important to continue studying more methods to produce PDMS-based devices on a larger scale which would further enable these devices to reach the market. Additionally, the currently available PDMS hydrophilic treatments need further developments and improvements as, most of the time, they do not last long. Hence, it is important to develop new methods or improve the existing ones in order to achieve a higher permanent hydrophilic feature for PDMS.

## Figures and Tables

**Table 1:** Typical properties of cured PDMS.

Property (Unity)	Result	References
Transmittance at range 390 nm to 780 nm (%)	75–92	[54,55]
Index of refraction	1.4	[56]
Thermal conductivity (W/m∙K)	0.2–0.27	[57,58]
Specific heat (kJ/kg∙K)	1.46	[56]
Dielectric strength (kV/mm)	19	[57]
Dielectric constant	2.3–2.8	[56]
Electrical conductivity (ohm∙m)	4 × 10^13^	[56]
Volume resistivity (ohm∙cm)	2.9 × 10^14^	[57]
Young’s modulus [kPa]	360–870	[59]
Poisson ratio	0.5	[60]
Tensile strength (MPa)	2.24–6.7	[56,57]
Hardness [Shore A]	41–43	[55,61]
Viscosity (Pa∙s)	3.5	[57]
Hydrophobicity—contact angle (°)	~108° ± 7°	[62]
Melting Point (°C)	−49.9 to −40	[63]

**Table 2:** Recommended curing times and temperatures to produce PDMS samples [57].

Temperature (°C)	Time
25	48 h
100	35 min
125	20 min
150	10 min

**Table 3:** Applications of PDMS-based microchips and respective motivations for using PDMS.

Application	PDMS Preparation	Motivations for Using PDMS	Reference
On-line sample pre-treatment and contactless conductivity detection	Mixing ratio—10:1, *w*/*w* Degassing time—20 min Curing temperature—80 °C Curing time—30 min Oxygen plasma treatment for 1–2 min	Low-cost, easy manufacture, suitability for mass production, transparency and elasticity.	[115]
Genetic analysis by functional integration of polymerase chain reaction (PCR) and capillary gel electrophoresis (CGE)	Mixing ratio—10:1, *w*/*w* Degassing time—15 min Curing temperature—65 °C Curing time—1 h Post-curing temperature—135 °C Post-curing time—15 min Hydrophilic treatment with HCl solution at 25 °C for 4 h	Low-cost, suitability for microscale moulding, high reproducibility on a micrometre scale, high gas permeability, low thermal conductivity and transparency.	[118]
Polymerase chain reaction (PCR)	Mixing ratio—10:1, *w*/*w* Curing temperature—95 °C Curing time—30 min	Low thermal conductivity, simple fabrication, low-cost, disposability, biocompatibility, irreversible bonding with glass and transparency.	[120]
Electrophoresis device for continuous on-line in vivo monitoring of micro dialysis samples	Mixing ratio—10.5:1.5, *w*/*w* 5 mm-thick layer curing temperature—90 °C 5 mm-thick layer curing time—25–30 min 1 mm-thick layer curing temperature–90 °C 1 mm-thick layer curing time—15–18 min Post-curing temperature—85 °C Post-curing time—overnight	Easy manufacture, good reproductivity and transparency.	[121]
Generation of temperature gradient	Mixing ratio—10:1, *w*/*w*	Low-cost, transparency, easy manufacture and low thermal conductivity	[122]

**Table 4:** Advantages and limitations of techniques used to fabricate microchannels.

Geometry	Method	Advantages	Limitations	Application	Reference
Rectangular	Soft lithography	Generation of precise, reproducible and versatile microchannels; Precise control of experimental parameters and accurate measurements; Inexpensive, simple and rapid method.	Different geometry from in vivo microvessels; Difficulties in achieving stable cell seeding at the corners of the channel.	Integration of confocal micro-PIV with a PDMS microchannel to obtain blood velocity profiles	[126]
Circular	Wire casting technique	Simple and inexpensive method; Possibility of fabricating microchannels with different diameters; No need for a clean room or specialized equipment.	It is not possible to generate well-defined complex structures, such as bifurcations.	In vitro hemodynamic studies	[127]
	Partially cured PDMS combined with thermal air expansion molding	Inexpensive and simple method; Possibility of fabricating multiple diameters of circular channel from 100 µm to 500 µm and different cross-sections.	It can be hard to fabricate a perfect circular channel.	Evaluate the clotting events in pathological vessels and testing device for antiplatelet and anticoagulant therapeutics	[128]
	Combination of soft lithography with the reflow phenomenon of a positive photoresist	Simple and efficient method; Possibility of fabricating microchannels with multiple diameters (from 100 µm to 400 µm) and various channel designs.	It can be hard to control the thickness of the photoresist, leading to a difficulty in generate perfect circular channels; Bonding the two semi-circular channels perfectly can be challenging.	This method allows endothelial cells culture, making this project suitable for drug screening and chemical/biological diagnostics	[90]
	Reshaping rectangular microchannels through polymerization of the liquid silicone oligomer around a gas steam	Ability of controlling the diameter from 40 µm to 100 µm; Possibility of fabricating constrictions.	Relatively complex and expensive method; Difficulty in controlling the exact diameter of the channel.	Mimic in vivo systems for cell flow studies	[124]

**Table 5:** Developments and applications of PDMS-based coatings.

Application	PDMS Preparation	Motivation for Using PDMS	Reference
Urethanes PDMS-based hybrid coating for metallic dental implants	Hybrid urethanesil (PDMSUr) synthesized by ring opening polymerization of a bis(cyclic carbonate) derived from PDMS. Curing temperature—60 °C Curing time—24 h	Create hydrophobic and smooth surfaces, with less adhesion of bacteria, capable of adhering to tissue cells such as fibroblasts and osteoblasts.	[38]
Tantalum oxide-PDMS hybrid coating for medical implants	Modified sol-gel synthesis method, Tantalum oxide-PDMS solutions (10%, *v*/*v*). Curing temperature—room temperature Curing time—15 min	Medical grade PDMS has functional groups to bind to reactive surfaces such as activated metals or polymers. Ability to create micrometer-thick coatings.	[39]
Bioactive CaO-SiO_2_-PDMS coatings	Sol-gel dip-coating method. The produced coatings were kept at room temperature for 24 h for gelation. Curing temperature—150 °C Curing time—24 h	Mechanical properties and elasticity of PDMS	[40]
PDMS-based coating for a bladder volume monitoring sensor	Mixing ratio—10:2 (*w*/*w*) Curing temperature—80 °C Curing time—2 h	Biocompatibility, 10:2 ratio to increase tensile strength and improve Young’s modulus	[41]
CuO-PDMS-SiO_2_ coatings	Mixing ratio—10:1 (*w*/*w*) Curing temperature—150 °C Curing time—90 min	Improved biocompatibility, corrosion resistance and antibacterial property	[42]

## Data Availability

Not applicable.
